# The effect of HIV infection and HCV viremia on inflammatory mediators and hepatic injury—The Women’s Interagency HIV Study

**DOI:** 10.1371/journal.pone.0181004

**Published:** 2017-09-13

**Authors:** Sheila M. Keating, Jennifer L. Dodge, Philip J. Norris, John Heitman, Stephen J. Gange, Audrey L. French, Marshall J. Glesby, Brian R. Edlin, Patricia S. Latham, Maria C. Villacres, Ruth M. Greenblatt, Marion G. Peters, the Women’s Interagency HIV Study

**Affiliations:** 1 Blood Systems Research Institute, San Francisco, California, United States of America; 2 Department of Laboratory Medicine, University of California San Francisco, California, United States of America; 3 Department of Surgery, UCSF, San Francisco, California, United States of America; 4 Department of Medicine, UCSF, San Francisco, California, United States of America; 5 Department of Epidemiology, Johns Hopkins University Bloomberg School of Public Health, Baltimore, Maryland, United States of America; 6 CORE Center, Stroger Hospital of Cook County, Chicago, Illinois, United States of America; 7 Department of Medicine, Division of Infectious Diseases, Weill Cornell Medical College, New York, New York, United States of America; 8 Department of Medicine, SUNY Downstate, Brooklyn, New York, United States of America; 9 Department of Pathology and Medicine, George Washington University Medical Center, Washington DC, United States of America; 10 Department of Pediatrics, Keck School of Medicine, University of Southern California, Los Angeles, California, United States of America; 11 Department of Pharmacology, UCSF, San Francisco, California, United States of America; University of Cincinnati College of Medicine, UNITED STATES

## Abstract

Hepatitis C virus infection induces inflammation and while it is believed that HIV co-infection enhances this response, HIV control may reduce inflammation and liver fibrosis in resolved or viremic HCV infection. Measurement of systemic biomarkers in co-infection could help define the mechanism of inflammation on fibrosis and determine if HIV control reduces liver pathology. A nested case-control study was performed to explore the relationship of systemic biomarkers of inflammation with liver fibrosis in HCV viremic and/or seropositive women with and without HIV infection. Serum cytokines, chemokines, growth factors and cell adhesion molecules were measured in HIV uninfected (HIV-, n = 18), ART-treated HIV-controlled (ARTc, n = 20), uncontrolled on anti-retroviral therapy (ARTuc, n = 21) and elite HIV controllers (Elite, n = 20). All were HCV seroreactive and had either resolved (HCV RNA-; <50IU/mL) or had chronic HCV infection (HCV RNA+). In HCV and HIV groups, aspartate aminotransferase to platelet ratio (APRI) was measured and compared to serum cytokines, chemokines, growth factors and cell adhesion molecules. APRI correlated with sVCAM, sICAM, IL-10, and IP-10 levels and inversely correlated with EGF, IL-17, TGF-α and MMP-9 levels. Collectively, all HCV RNA+ subjects had higher sVCAM, sICAM and IP-10 compared to HCV RNA-. In the ART-treated HCV RNA+ groups, TNF-α, GRO, IP-10, MCP-1 and MDC were higher than HIV-, Elite or both. In ARTuc, FGF-2, MPO, soluble E-selectin, MMP-9, IL-17, GM-CSF and TGF-α are lower than HIV-, Elite or both. Differential expression of soluble markers may reveal mechanisms of pathogenesis or possibly reduction of fibrosis in HCV/HIV co-infection.

## Introduction

Hepatic disease is a leading cause of significant morbidity and death among HIV infected persons in the US; 15–30% of HIV-infected individuals are coinfected with hepatitis C virus (HCV)[1–4] and this is associated with metabolic and cardiovascular complications in addition to other inflammation induced comorbidities. Individually, HIV and HCV infections increase expression of inflammatory cytokines and chemokines [5–7]. These factors have been found to be associated with long-term morbidity in HIV infection or chronic hepatitis [7–11]. Additionally, HIV infection with HCV viral hepatitis more than triples the rate of liver disease, liver failure, and liver-related death [1]. The mechanisms causing accelerated disease with co-infection are not well understood but there is evidence that HIV infection increases morbidity in HCV co-infected individuals [12]. HIV-induced immune perturbation, including CD4 cell loss, generalized inflammation and trafficking of activated immune cells to the liver in HCV infection likely also results in greater tissue damage and fibrosis [13,14].

Although an HIV-specific host immune response is required to control HIV viremia, it may also result in broad and non-specific immune activation and an array of tissue injuries including hepatic fibrosis. Alternatively, HIV suppression may reduce non-specific inflammation and reduce bystander inflammation-induced fibrosis. Individually, HIV and HCV immune activation induces expression of inflammatory cytokines (e.g. TNF-α and IL-1β [15,16]) and chemokines (IP-10, MCP-1, MIG and ITAC [13,17–20]) directing cellular immune responses to sites of infection. Enhanced expression of chemokine receptors on lymphocytes (e.g. CXCR3 [21–24]) increases the transit of immune cells to sites of infection; meanwhile, higher expression of cellular adhesion molecules increases cell trafficking through the vascular endothelium to the site of infection [18,25–27].

In order to further investigate the effect of different states of HIV infection on hepatic injury in chronic HCV, we measured soluble biomarkers in HIV- and HIV+ women with HCV. We hypothesized that lower inflammatory responses and less liver fibrosis would be found in HCV+ women with controlled HIV replication including elite controllers and ART-treated with viral suppression (ARTc) than in ART-treated women with uncontrolled HIV replication (ARTuc). This would describe a distinct biomarker profile in relation to stage of liver diseases and elucidate the clinically relevant biomarkers, and mechanisms of hepatic pathogenesis in HIV/HCV co-infection.

## Materials and methods

### HCV antibody positive women

This study was limited to HCV serologically reactive women enrolled in the Women’s Interagency HIV Study (WIHS). The details of this prospective, multi-center, longitudinal cohort NIH study have been published previously [28]. Briefly, the WIHS enrolled 3766 adult women in two recruitment periods (1994–1995 and 2001–2002) at six clinical sites across the United States. Women were either infected with HIV or at high risk for acquiring the infection. Overall, 32% of HIV-seropositive and HIV-seronegative women had HCV antibody at enrollment. All participants gave written, informed consent for the WIHS as approved by all Institutional Review Boards (IRB) of the participating institutions and this study was approved by UCSF IRB. Every 6 months, participants undergo a comprehensive physical examination, provide biological specimens for CD4 cell count and HIV-RNA viral load determination, and complete an interviewer-administered questionnaire that collects information on demographics, health history, alcohol consumption and medication use. Heavy alcohol was defined as 14 or more standard drinks per week.

Serologic reactivity to HCV was determined using a second-generation or third-generation enzyme immunoassay (EIA, Ortho-Diagnostic Systems); women with positive EIA tests also were assessed for HCV-RNA by RT-PCR (COBAS Amplicor HCV Detection Kit, Roche Diagnostic Systems). HIV-RNA was measured using the isothermal nucleic acid sequence-based amplification (NASBA/Nuclisens) method (bioMerieux) with a detection limit of 80 copies/mL. All women were hepatitis B surface Ag negative.

The study population was comprised of homogenous, well characterized HIV phenotypes (stable for at least 1.5 years) stratified by serum HCV RNA positive (HCV RNA+) or negative (HCV RNA-<50IU/mL). Women were matched by age, race/ethnicity, and HCV RNA status within the following groups: Elite controllers (EC) were defined as ART-naïve, CD4 cell count never below 500, HIV RNA < 80 copies /mL. Groups were limited to the number of EC women within the WIHS where there were samples were available to study. These were very carefully selected groups based on the number of Elite controllers. ART treated individuals with controlled infection (ARTc) were HIV women currently on potent ART with viral control, with CD4 cell count > 350 cells/ml, HIV RNA< 80 copies/ml. ART treated with uncontrolled infection (ARTuc) were ART-treated women but without HIV uncontrolled having HIV RNA ≥80 copies/ml. HIV uninfected were HIV negative by EIA. No participants in this study were HBV positive.

### Assessment of liver fibrosis

The serum marker of liver fibrosis were aspartate aminotransferase (AST) to peripheral blood platelet count ratio (APRI) [29] and FIB-4, both of which have been validated in HIV-HCV coinfected women [30]. Using a laboratory upper limit of normal AST of 40 U/l, severe fibrosis is defined by APRI >1.5, FIB-4 >3.25 and mild/no fibrosis as APRI score <0.5 and FIB-4 <1.5 [31].

### Measurement of soluble immunologic markers

Plasma samples were assayed using a high-sensitivity Milliplex kit (Millipore) with antibody-coated beads for detection of GM-CSF, interferon (IFN)-γ, interleukin(IL)-10, IL-12(p70), IL-13, IL-1, IL-2, IL-4, IL-5, IL-6, IL-7, IL-8, and tumor necrosis factor (TNF)- α;a standard-sensitivity Milliplex Map kit (Millipore) for epidermal growth factor (EGF), eotaxin, fibroblast growth factor (FGF)-2, fractalkine, Flt-3 ligand, GRO, G-CSF, IFN-α2, IL-1 α, IL-1Rα, IL-3, IL-9, IL-12(p40), IL-15, IL-17, IFN-γ-induced protein (IP)-10, monocyte chemotactic protein (MCP)-1, MCP-3, macrophage-derived chemokine (MDC), macrophage inflammatory protein (MIP)-1α, MIP-1 β, sIL-2 receptor α (Rα), transforming growth factor (TGF)- α, TNF- β, vascular endothelial growth factor (VEGF) and soluble CD40 ligand (sCD40L) and a cardiovascular panel including soluble E-selectin, soluble vascular cell adhesion molecule (s-VCAM), soluble intracellular adhesion molecule (s-ICAM), matrix metalloproteinase-9 (MMP-9), myeloperoxidase (MPO), total plasminogen activator inhibitor (tPAI). Testing was performed following the manufacturer’s protocols. Standard curves and samples were tested in duplicate. The standard curve detection ranged from 2–25000 ng/mL for the cardiovascular panel, 1.3–2000 pg/mL for the high sensitivity panel, and 3.2–10,000 pg/mL for the standard sensitivity panel. The values that were out of range low were assigned a value half the lowest standard. The values that were out of range high were assigned a value twice the highest value in the set. Results were acquired on a Labscan 200 analyzer (Luminex) using Bio-Plex manager software v6.1 (Bio-Rad), and study plates were compiled using Data Pro (Bio-Rad). Data were exported to Prism (Graphpad) for graphing and SAS for analysis.

### Statistical analysis

Demographic and clinical characteristics were described with means (standard deviations), medians (interquartile ranges) and frequencies (percent) stratified by HIV clinical subgroups and presence or absence of HCV viremia. APRI, chemokines, 45 cytokines and cell adhesion molecules were measured. Twelve soluble markers received 50% or fewer detectable results for a given cytokine were excluded from further analysis; these included IL-4, IL-12p70, IL-13, IFNα2, IL-1α, IL-1ra, IL-3, IL-9, IL-12-p40, IL-15, IL-2R α and TNF- β. One marker, soluble CD40L, was out of range high in more than 50% of the results and was excluded from further analysis. Cytokines compared by HCV viremia status using the t test and by HIV clinical subgroups using the Tukey method to account for multiple pairwise comparisons. Variables were transformed (log_10_ or square root) to achieve normality, as needed. Correlation of CD4 count, chemokines, cytokines and cell adhesion molecules with APRI were measured with Spearman’s rank correlation coefficients for the total population and within HCV RNA+ HIV clinical subgroups. Given the small sample size within subgroups, non-parametric correlations were used to minimize the influence of potential outliers. To control for the expected proportion of incorrectly rejected null hypotheses, p-values for comparisons among biological markers were adjusted into FDR (False Discovery Rates) by the Benjamini and Hochberg controlling procedure [32], a commonly used method for analysis of large sets of biological data. While statistical significance was defined as p<0.05 and reported for all comparisons, due to the small sample size and discovery nature of this study, the FDR are also reported to describe the strength of the findings. Data analysis and graphing were performed by SAS version 9.3 (Cary, NC) and Prism (GraphPad), respectively.

## Results

### Patient population

Seventy-nine HCV antibody positive women were identified and grouped as follows (Table 1): Elites (n = 20), controlled HIV on ART (ARTc; n = 20); uncontrolled on ART (ARTuc; n = 21) and HIV uninfected (n = 18). Mean age (SD) was 49 (±7) years ranging from 35.6–62.9 years, mean CD4 cell count among HIV infected women was 681 cells/μL (±399), and 54% of women reported being African American (AA). There were no differences in age and race between the subgroups. To control for variations in all biomarkers for effects of alcohol consumption, we compared the high (n = 20) and low alcohol consumers and found that there were no differences in the biomarkers by alcohol consumption (p>0.05 for all cytokines). There were 9 individuals who had received HCV treatment in the past and all failed to clear HCV. All individuals who cleared HCV did so spontaneously, without treatment, prior to entering WIHS.

**Table 1:** Demographics.

	HIVneg		Elite		ARTc		ARTuc	
HCV RNA	Neg (n = 8)	Pos (n = 10)	Neg (n = 9)	Pos (n = 11)	Neg (n = 10)	Pos (n = 10)	Neg (n = 10)	Pos (n = 11)
Age, years (mean ±SD)	51.3 ±4.8	45.5 ±7.5	46.9 ±7.7	47.6 ±6.4	50.9 ±7.2	51.0 ±4.1	48.8 ±7.8	48.6 ±5.9
Race, AA (% total)	50%	50%	33%	64%	60%	70%	50%	55%
HIV RNA; log10 copies/mL (mean ±SD)			ND	ND	ND	ND	3.61 ±1.01	2.98 ±0.78
CD4 count*; cells/μL (mean ±SD)	1123 ±336	910 ±254	897 ±255	918 ±429	996 ±336	722 ±226	297 ±171	294 ±169
HCV RNA; log10 copies/mL (mean ±SD)	ND	5.49 ±1.06	ND	5.79 ±0.44	ND	6.14 ±0.93	ND	6.24 ±0.31
APRI	0.17 ±0.05	0.48 ±0.38	0.38 ±0.56	0.46 ±0.25	0.37 ±0.17	0.78 ±0.50	0.17 ±0.07	1.36 ±1.62
Serum AST log10 (mean ±SD)	1.22 ±0.11	1.55 ±0.21	1.27 ±0.10	1.53 ±0.14	1.46 ±0.15	1.64 ±0.18	1.19 ±0.08	1.68 ±0.27
Serum ALT; log10 (mean ±SD)	1.09 ±0.16	1.49 ±0.18	1.13 ±0.17	1.52 ±0.17	1.35 ±0.25	1.52 ±0.18	1.11 ±0.16	1.66 ±0.26
Platelet count (x 10^3^/microliter) (mean ±SD)	260.2 ±44.8	237.7 ±52.5	250.4 ±118.7	230.3 ±69.5	231.6 ±62.7	208.1 ±62.3	268.1 ±58.5	219.2 ±130.1

Abbreviations: SD = standard deviation; ND = not detectable (<80 RNA copies/mL for HIV and <80 RNA copies/mL for HCV); AA = African American.

*CD4 differed between HCV RNA negative versus positive (ARTc p = 0.04) and by HIV subgroup (ARTuc p<0.001).

### Correlation of chemokines, cytokines and cell adhesion molecules levels with liver fibrosis as measured by APRI

**Fig 1:**
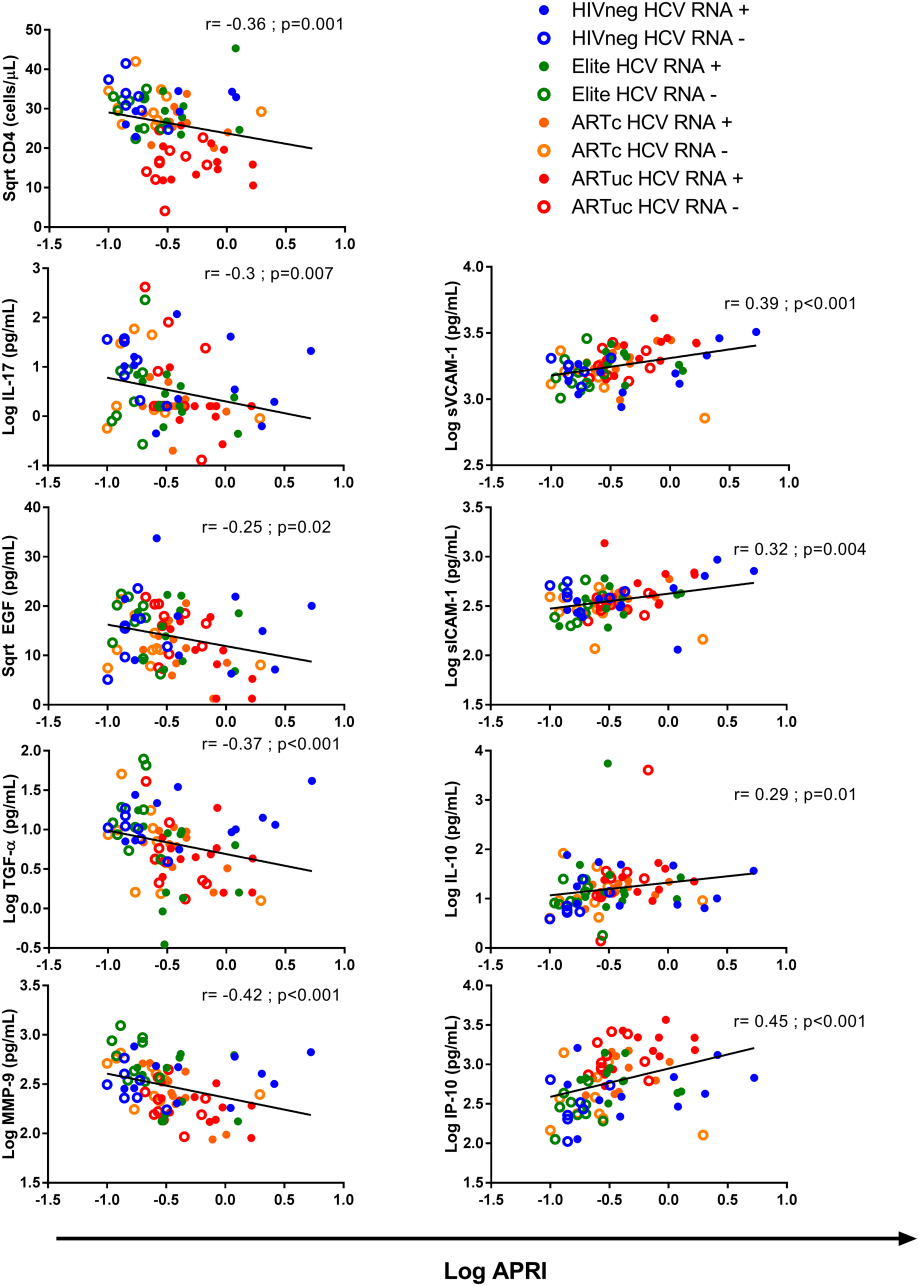
Cytokines and chemokines correlate with liver fibrosis. Cytokines, chemokines and cell adhesion molecules were evaluated for correlation with APRI, a marker of liver fibrosis. The subgroups were broken down by color: HIV Neg (blue), Elite (green), HIV uncontrolled (ARTuc; red) and HIV controlled (ARTc; orange), and by HCV RNA status: positive (closed circles) and negative (open circles).

In another study based on a similar sample set in WIHS [14], it has been previously published that APRI was higher in HCV RNA+ women compared to HCV RNA-. Furthermore, this pattern of lower APRI in HCV non-viremic and higher APRI in HCV viremic women was observed within each HIV clinical group. In Elite, APRI was higher in HCV RNA+ compared to RNA-. APRI was also higher in HCV RNA+ compared to HCV RNA- subjects within each of the other clinical groups (ARTc, ARTuc and HIV-). To identify soluble biomarkers and inflammatory responses involved in hepatic injury, we focused our investigations on 32 markers including cytokines, chemokines and cell adhesion molecules (45 markers were measured, 32 markers were detectable; Table 2). We found that sVCAM (r = 0.38; p<0.001; FDR = 0.008), sICAM (r = 0.32; p = 0.004; FDR = 0.03). IL-10 (r = 0.29; p = 0.01, FDR = 0.07) and IP-10 (r = 0.45; p<0.001; FDR = 0.002) were positively correlated with APRI while IL-17 (r = -0.3; p = 0.007; FDR = 0.05), EGF (r = -0.25; p = 0.02; FDR = 0.13), TGF- α (r = -0.37; p<0.001; FDR = 0.009) and MMP-9 levels (r = -0.41; p<0.001; FDR = 0.002) were inversely correlated with the APRI (Fig 1). We found similar results with FIB-4 (S1 Fig). When stratifying the HCV RNA+ group by HIV clinical subgroups, sVCAM levels correlated with APRI in ARTuc (r = 0.66, p = 0.03; FDR = 0.3), while correlation of sICAM with APRI was found only in the ARTc group (r = 0.72, p = 0.02; FDR = 0.2). None of the soluble markers correlated with APRI in the elite controller group.

**Table 2:** Biomarker measurements in all HCV and HIV groups.

	HIV negative		Elite RNA Neg		ART Controlled		ART Uncontrolled	
	HCV RNA Neg	HCV RNA Pos	HCV RNA Neg	HCV RNA Pos	HCV RNA Neg	HCV RNA Pos	HCV RNA Neg	HCV RNA Pos
	n = 8	n = 10	n = 9	n = 11	n = 10	n = 10	n = 10	n = 11
**APRI**^**^**^	0.17 (0.14–0.19)	0.755 (0.24–2.18)	0.17 (0.13–0.21)	0.32 (0.29–0.43)	0.245 (0.13–0.29)	0.37 (0.31–0.54)	0.285 (0.27–0.50)	0.74 (0.34–0.95)
**IL-1α**	0.3 (0.1–0.6)	2.6 (0.2–4.4)	0.1 (0.1–0.5)	0.3 (0.1–3.7)	0.1 (0.1–0.6)	0.3 (0.1–2.9)	0.3 (0.1–1)	0.1 (0.1–1)
**IL-2**	0.3 (0.1–1.5)	2.1 (0.2–7.9)	0.1 (0.1–0.8)	2.1 (0.2–5.9)	0.2 (0.1–0.4)	0.3 (0.2–1.7)	0.4 (0.1–1.1)	0.2 (0.1–0.9)
**IL-5**	0.3 (0.1–0.5)	0.4 (0.1–0.7)	0.2 (0.1–0.4)	0.4 (0.2–0.8)	0.4 (0.1–0.9)	0.4 (0.1–0.6)	0.4 (0.2–0.6)	0.2 (0.2–0.5)
**IL-6**	12.2 (5.5–22.2)	11.5 (7.4–23.4)	6.5 (2.7–21.6)	4.1 (2.7–23)	6.5 (1.5–13)	7.7 (1–13.7)	4.8 (3.7–10.1)	4.9 (2.3–6)
**IL-7**	4.5 (2.2–7.3)	8.4 (3.5–11.8)	5.7 (3.1–8.6)	3.5 (1.7–9.1)	10.5 (4.2–15.7)	7.7 (6.2–8.9)	15.3 (6.6–26.6)	11.1 (5.4–19.8)
**IL-8**	21.2 (7.2–75.4)	23 (7.6–119.4)	43.6 (17.7–177.1)	32.2 (9.3–123.2)	18.7 (7.1–35.1)	22.1 (10.8–55.6)	17.8 (6.9–42)	21.3 (14–67)
**IL-10**	6.5 (5.4–12.1)	27.2 (7.5–50.9)	8.9 (8–20.8)	14.1 (10.6–34.9)	9.5 (7.4–24.5)	18.2 (13.3–22.2)	21.5 (11.5–34.9)	22.4 (12.7–27.3)
**IFN-γ**	0.1 (0.1–1.1)	1.1 (0.1–8.2)	1.4 (0.1–2)	1.5 (0.8–7.1)	0.1 (0.1–1.6)	0.3 (0.1–6.4)	0.1 (0.1–1.5)	0.1 (0.1–1)
**Gm-CSF**	0.4 (0.1–1.6)	3.8 (1.5–6)	0.3 (0.2–1)	0.5 (0.1–13.7)	0.2 (0.1–0.7)	1.2 (0.1–4.2)	0.1 (0.1–1.1)	0.1 (0.1–2.8)
**TNF-α**	8.7 (5.5–12.8)	7.1 (4.5–9.7)	8.1 (5–13)	7.1 (5.4–11.6)	10.6 (6.7–17.4)	11.1 (8.8–15.6)	14.6 (10–18.8)	14.7 (9.8–19.4)
**sE-Selectin**	47.8 (36.7–78.2)	57.6 (46.6–94.6)	59.6 (39.3–64.6)	76.7 (55.9–104.2)	58.4 (36.2–66.7)	52.8 (39.1–64.8)	45.5 (26.1–59.2)	36.2 (30.1–61.6)
**sVCAM-1**	1697 (1313–1984)	1582 (1263–2328)	1449 (1281–1793)	1857 (1545–2144)	1628 (1281–1865)	1984 (1697–2576)	1728 (1488–2355)	2561 (1953–2712)
**sICAM-1**	386.5 (271.1–492.8)	364.9 (303.1–659.8)	267.4 (207.4–448.8)	395.5 (259.9–459.9)	292.3 (237.7–407)	364.9 (327.9–403.1)	320.5 (278.2–354.6)	455.3 (388.2–670.3)
**MMP-9**	300.1 (229.2–388.9)	436.8 (263.5–617.3)	613.8 (357.6–905.3)	301 (132.4–503.3)	376.7 (232.8–530.5)	249.1 (173.1–445.9)	194.6 (150.7–290.1)	192 (137.7–234)
**MPO**	140.7 (96.2–746.9)	482.7 (285.5–728.3)	868.2 (483.6–2584)	293.9 (127.2–776.2)	253 (140.8–431.3)	174.3 (74.7–274.6)	112.9 (74.6–311.9)	99 (85.4–227.9)
**total PAI-1**	122.7 (103.2–156.5)	150.3 (117.1–183.4)	114.6 (102.5–161.5)	115.3 (91.9–126.8)	163.8 (116.3–217.5)	124.2 (105.7–161.1)	129.6 (99.2–198.9)	105.6 (93.1–186.2)
**EGF**	189.9 (84.7–293.3)	318.2 (88.3–465.4)	311 (123.2–443.2)	223 (71.8–386.4)	127.4 (108.9–292.6)	98.3 (55.1–204.8)	297 (132.6–416.6)	121.5 (27.5–256.7)
**Eotaxin**	86.8 (64.9–102.8)	107.8 (66.6–157.6)	117.6 (43.8–191.7)	91.5 (44–137)	193.6 (82.5–254.5)	152.1 (106.9–217.6)	165.7 (72.4–256.4)	108.6 (90.2–215)
**FGF-2**	41.5 (15.7–63.6)	89.4 (41.4–132.5)	18.2 (1.6–50.5)	44.9 (22.9–70.3)	6.8 (1.6–31.7)	1.6 (1.6–79.1)	1.6 (1.6–63.6)	1.6 (1.6–111.9)
**Fit-3 Ligand**	24.1 (1.6–48.9)	4.2 (1.6–115.1)	7.2 (1.6–79.2)	1.7 (1.6–47.8)	1.6 (1.6–103.4)	3 (1.6–27)	19 (1.6–24.7)	1.6 (1.6–22.5)
**Fractalkine**	81.2 (30.2–118)	52.6 (1.6–105.2)	1.6 (1.6–215.1)	33.8 (1.6–113)	1.6 (1.6–12.9)	67.1 (14.2–161.5)	4 (1.6–104.1)	1.6 (1.6–52.4)
**G-CSF**	43.4 (32.8–60.6)	47.2 (38.1–155.4)	45.8 (19.3–118.3)	34.7 (9.7–46)	32.6 (27.2–63.1)	37.7 (21.3–64.9)	37 (21.9–75.6)	30.2 (19.8–43.2)
**GRO**	2777 (1586–3696)	1686 (1085–2587)	2198 (1146–2413)	1412 (675.7–1995)	1874 (1494–3835)	1945 (1521–2809)	2587 (1769–3233)	2721 (2030–3026)
**IL-17**	14.9 (3.3–35.3)	7 (1.7–26.2)	2 (1–8.6)	2.7 (1.1–5.6)	1.6 (1.2–33.9)	1.6 (1.6–3)	1.6 (1.6–38.4)	1.6 (1–1.6)
**IP-10**	249.9 (135.7–491.5)	490.5 (336.9–850.2)	287.7 (210.4–383.3)	655.7 (449.2–1025)	304.1 (191.7–789.5)	788.7 (442.1–1316)	1066 (836.5–2029)	1522 (867.9–2197)
**MCP-1**	371.6 (264.6–660.3)	431 (244.1–675.9)	545.5 (369.4–1050)	414.8 (274.1–592.9)	776.8 (407.4–1355)	825.8 (612.9–1158)	692.4 (512.7–1491)	658.7 (399.4–832.4)
**MCP-3**	18.3 (10.4–31.3)	43.6 (21.1–75.1)	7 (1.6–30.5)	11.8 (3–25.2)	1.6 (1.6–15.6)	28.1 (1.6–53.9)	4.8 (1.6–23.7)	7 (1.6–18.3)
**MDC**	3790 (3344–4557)	3770 (1972–4768)	2469 (1292–5558)	1560 (817.9–3023)	3884 (2607–4135)	3467 (2775–4716)	3867 (2250–4893)	2927 (2431–3902)
**MIP-1α**	64.8 (48.7–147.7)	100.7 (46–150.6)	71.9 (15.4–734.9)	29.6 (13.8–95.5)	41.9 (5.7–117.8)	81 (31.1–148.9)	108.1 (26.8–861.9)	61.8 (30.3–129)
**MIP-1β**	78.7 (38.8–169.9)	105.6 (81–309.5)	125.1 (31.6–285.7)	50.3 (30.4–149.4)	53.2 (44.4–95.4)	91.5 (62.6–125.5)	73.3 (49.3–214.3)	61 (22.5–104.3)
**TGF-α**	10.5 (7.4–14.1)	12.9 (8.8–29.4)	12.3 (7.1–42.4)	4 (1.3–10)	5.7 (1.6–10)	6.8 (3.4–9.8)	4.2 (2.2–10.4)	4.5 (2.6–5.9)
**VEGF**	394.7 (301.9–546.8)	334.8 (188.5–879.4)	244.2 (18.4–628.3)	129.4 (50.7–271.7)	652 (169.5–957.4)	296.9 (218.8–536.3)	439.9 (256.4–1717)	332.4 (193.8–613.3)

### Soluble cytokines, chemokines and cell adhesion molecules are associated with HCV viremia

**Fig 2:**
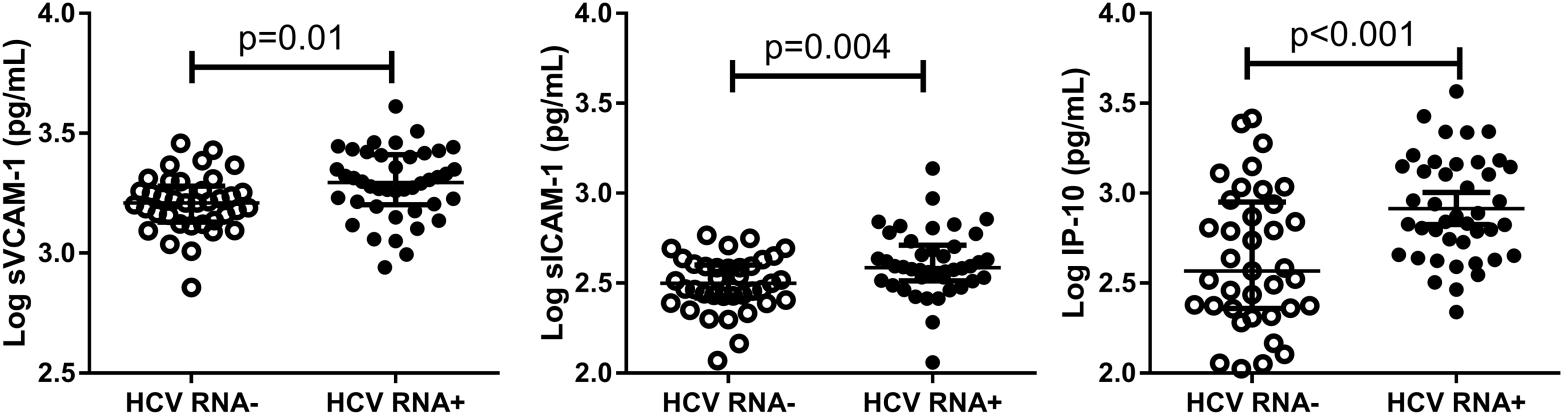
VCAM-1, ICAM-1 and IP-10 as soluble markers of HCV replication. Cytokines, chemokines and cell adhesion molecules were compared across HCV RNA status. The bars represent the median and interquartile ranges.

**Fig 3:**
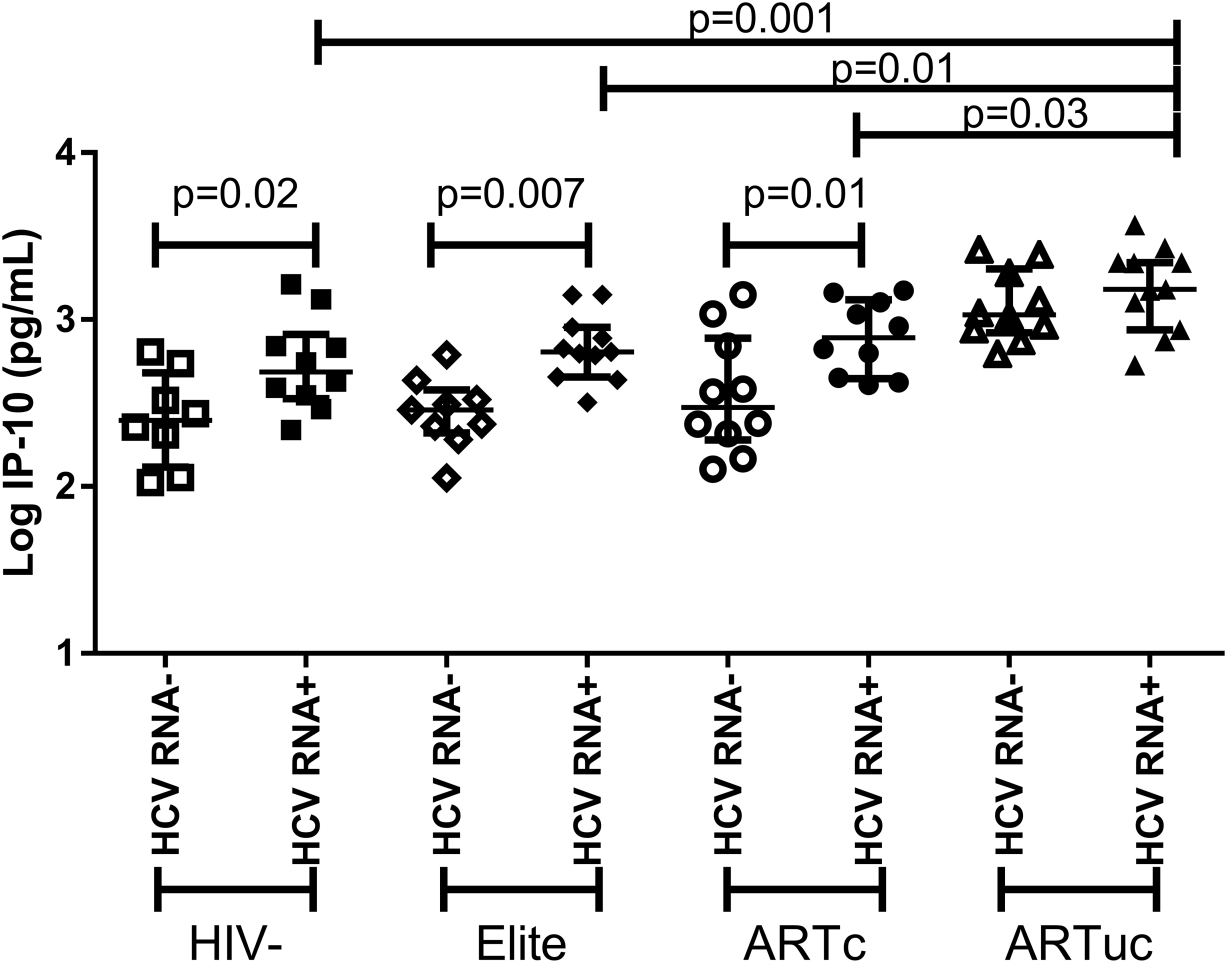
Elevations in IP-10 in HIV-HCV co-infection. Significant elevations of IP-10 were observed in all HCV RNA+ groups compared to HCVRNA- except for ARTuc. The bars represent the median and interquartile ranges.

Previous studies demonstrated that inflammatory liver diseases increase cell adhesion molecules, chemokine and cytokine expression [13,23]. In HCV mono-infection viremia increases cell adhesion molecules and chemokines directing cellular immune responses to the liver [33]. We found that HCV RNA+ status alone was associated with higher sVCAM (p = 0.01; FDR = 0.15), sICAM (p = 0.004; FDR = 0.1) and IP-10 levels (p = 0.001; FDR = 0.02) (Fig 2) compared to HCV RNA- status. In the HIV positive and negative subgroups, HCV RNA+ IP-10 levels were higher in ARTc, elite controller and HIV uninfected women compared to the corresponding HCV RNA- groups (Fig 3). Among ARTuc women, no difference in IP-10 by HCV RNA status was detected (p = 0.34; FDR = 0.7) and IP-10 levels were significantly elevated compared to HCV viremic ARTc, elite controller and HIV uninfected women.

**Fig 4:**
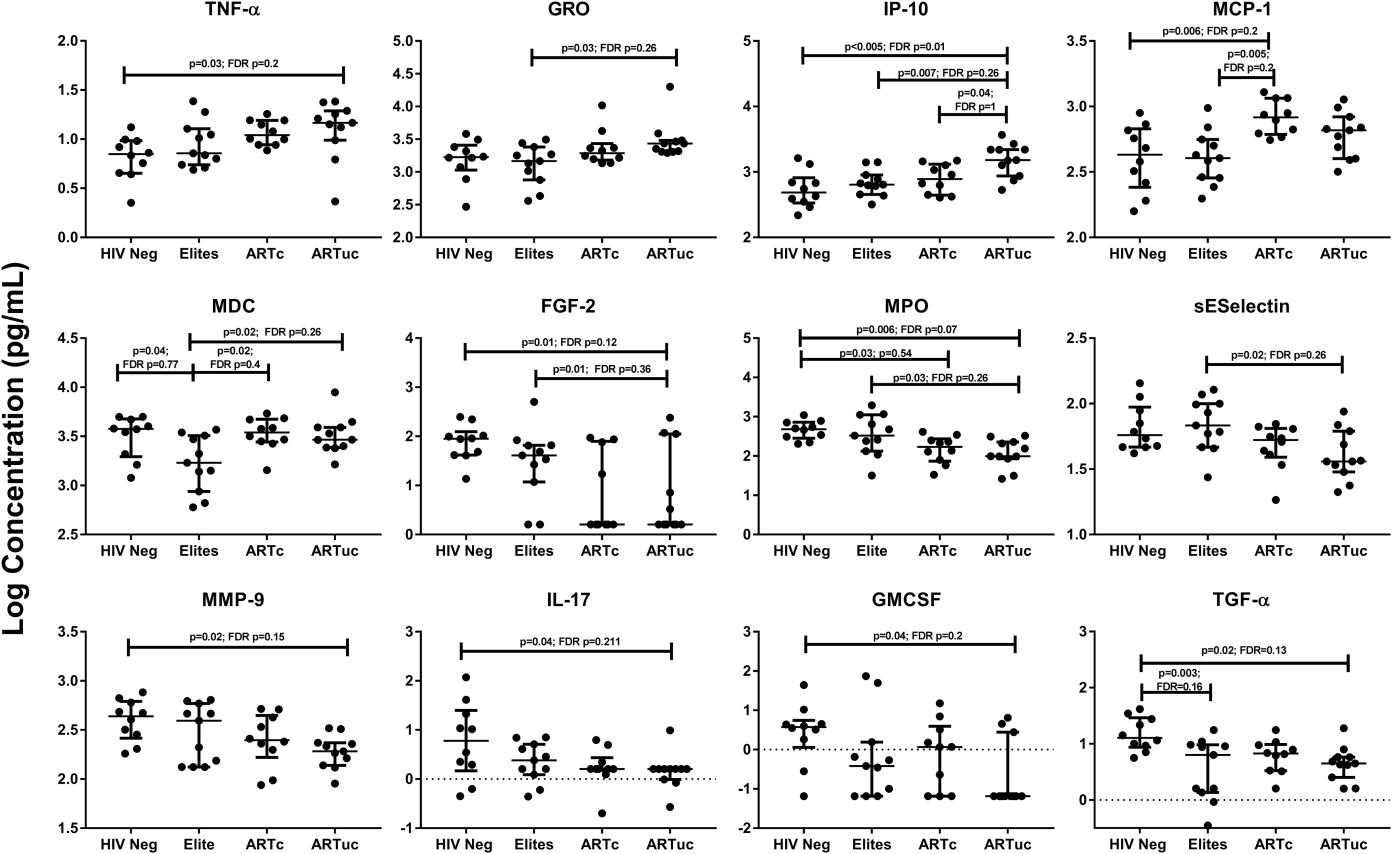
Differential expression of soluble immune mediators in HCV RNA+ individuals. The HCV RNA+ HIV negative and HIV positive groups were compared for all cytokines measured. Groups with significant cytokine differences are identified as p<0.05; a false discovery rate adjustment for multiple comparisons was performed (Benjamini Hochberg) all values are presented.

Cytokine concentrations were compared among the HCV+ HIV negative and positive groups. In HIV+ ART-treated groups, inflammatory cytokines and chemokines (TNF-α, GRO, IP-10 and MDC) were higher compared to the other groups. Even in treated and suppressed infection, chemokines MCP-1 and MDC were significantly elevated compared to the other groups. Compared to ART-treated individuals, higher concentrations in the HIV negative or Elite were found in some soluble biomarkers including FGF-2, MPO, soluble E-selectin, MMP-9, IL-17, GM-CSF and TGF-α(all p-values p<0.05; results in Table 3 and Fig 4).

**Table 3:** HCV RNA+ cytokine comparison in HIV negative and HIV positive groups.

p-values (FDR)	HIVneg vs Elite	HIVneg vs ARTc	HIVneg vs ARTuc	Elite VS ARTc	Elite vs ARTuc	ARTuc vs. ARTc
**GM-CSF**			0.04 (0.21)			
**TNF-α**			0.03 (0.19)			
**sE-Selectin**					0.03 (0.26)	
**MMP-9**			0.02 (0.15)			
**MPO**		0.04 (0.54)	0.006 (0.07)		0.03 (0.26)	
**FGF-2**		0.015 (0.36)	0.013 (0.12)			
**GRO**					0.03 (0.26)	
**IL-17**			0.04 (0.21)			
**IP-10**			0.0006 (0.1)		0.007 (0.26)	0.04 (1)
**MCP-1**		0.005 (0.27)		0.005 (0.23)		
**MDC**	0.03 (0.77)			0.02 (0.42)	0.02 (0.26)	
**TGF-α**	0.003 (0.16)		0.02 (0.13)			

## Discussion

Liver fibrosis, as measured by a high APRI or FIB-4 score, was associated with an increase in a number of inflammatory mediators including sICAM, sVCAM and IP-10 in women with chronic hepatitis C (HCV RNA+) compared to HCV RNA- women. One chemokine in particular, IP-10, an interferon-induced protein that has been implicated in liver fibrosis [34,35] and lower ability to clear infection [36], was elevated in HCV RNA+ women even among those women with suppressed HIV replication (elite controllers and ART responders). The very high IP-10 levels seen in the women with uncontrolled HIV replication dominate the relatively weaker IP-10 induction driven by HCV RNA. Other studies have shown increased hepatic disease and greater liver-related mortality in HIV-infected individuals with HCV co-infection [37,38], and our study identifies several pro-inflammatory markers potentially mechanistic associated with increased liver pathology. While ART control and/or elite control was associated with reduced IP-10, the inflammatory effects of HIV infection persisted when compared to HIV uninfected women [7].

The mechanisms underlying increased hepatic injury during HIV/HCV co-infection are not well understood. Strong anti-HCV cellular and antibody responses are induced during acute infection, and while in some cases this may result in resolved infection, in other cases the HCV infection persists [39,40]. There is evidence that cytokine and chemokine production, increased cell adhesion molecule expression, and cellular infiltration to the sites of hepatic infection contribute to tissue damage and fibrosis in HIV/HCV co-infected subjects [23]. A decrease in IFN-γ producing T cells show a reduction in anti-viral immune responses in the liver and reduced concentrations of regulatory T cells and elevated levels of chemokines and monocytes trafficking to the liver may promote liver fibrosis [41–43]. Previous studies have shown elevations in sVCAM-1 and sICAM-1 in advanced fibrosis and as predictors of liver-related events [44,45]. Our current work would support this hypothesis, showing the markers associated with fibrosis in this study were pro-inflammatory chemokines (IP-10), cytokines (IL-10) or were soluble cell adhesion molecules (sVCAM-1, sICAM-1). In line with this elevated inflammation with uncontrolled HIV replication we found significantly higher levels of TNF- α, IP-10 and GRO.

A few factors were found to be negatively correlated with APRI including IL-17, EGF, TGF-α and MMP-9 levels. Expression of these factors may result in a protective effect and reduced hepatic injury. Although many reports on the mechanisms of fibrosis indicate a link between pro-inflammatory mediators, such as IL-17, and hepatic injury and fibrosis, we found an inverse correlation between IL-17 and APRI score (r = -0.25; p = 0.02) with no relationship between IL-17 and CD4 count (r = 0.11, p = 0.3). Studies have shown that IL-17 exacerbates hepatic fibrosis in mice; however, co-expression of IL-17 and IL-22 has also been shown to ameliorate fibrosis [46]. This is further supported by the finding in another murine study that IL-22 induces senescence in hepatic stellate cells, thereby reducing fibrosis [47], which remains to be investigated since IL-22 was not assessed in our study. We found that EGF, MMP-9 and TGF- α levels were inversely correlated with APRI; in the HIVuc group, we also found significantly lower levels of FGF-2, MMP-9 and MPO compared to the other groups. EGF and TGF-α have a role in the regenerative response in the liver and EGF family members have been shown to inhibit TGF-β induced hepatic stellate cell activation and fibrosis induction [48–50]. Interestingly, TGF-α induces expression of MMPs which are involved in fibrinolysis [51]. In a murine model, after Flt3L-expressing B16 melanoma cells were injected into mice, it induced dendritic cell expression of MMP-9, which in turn reduced fibrosis [52]. Further studies investigating the expression of these markers on would be important in elucidating the mechanisms of protection from fibrosis.

After comparing the cytokines by HIV positive and negative groups, two different profiles of responses become evident. There is a subset of cytokines that shows higher concentrations in the ART-treated HCV+ groups (TNF-α, GRO, IP-10 and MDC) and a subset that shows lower concentrations in ART-treated groups (MCP-1, MDC, FGF-2, MPO, sE-Selectin, MMP-9, IL-17, GM-CSF and TGFα); although some differences were seen in both ART-treated groups, the differences were most evident in the group with uncontrolled HIV. As seen by earlier analysis, IP-10 in the HCV+ ART-treated groups was also found to be significantly correlated with increased fibrosis. Similarly, IL-17, TGF-α and MMP-9 were higher in the HIV negative and Elite groups and correlated with lower fibrosis. A number of the p-values were higher after correcting for false discovery rate and this may be due to the small sample size of the study groups. Regardless, the discovery of these markers is important in understanding the mechanism of pathogenesis in HIV and HCV co-infection and validation of these markers in larger, more targeted studies of these biomarkers will be required.

There are certain limitations to our study. Given the stringent eligibility criteria for inclusion in these analyses, sample size was limited by the number of available elite controllers; other groups were selected to achieve similar age and race distributions. Although small, our HIV groups were carefully selected and represented extremes of control or lack of control of HIV infection. These extreme HIV groups have not been studied in HCV viremic and non viremic women and show that both HIV and HCV status are important in immune responses. Our marker of fibrosis, APRI, was calculated from available clinical information was used as a surrogate for liver fibrosis and was highly correlated with the alternative marker for fibrosis, FIB-4. Both of these markers have been validated in WIHS and other studies and shown to be independently associated with all-cause mortality in HCV/HIV phenotypic groups (elite, ART controlled, ART uncontrolled, and HIV negative). Spearman correlation coefficients were 0.83 (p = 0.002), 0.83 (p = 0.003), 0.99 (p<0.001), and 0.89 (p<0.001), respectively [30,53–55]. We found similar results in the analysis of fibrosis and soluble markers (Fig 1 and S1 Fig). Therefore, we proceeded with APRI throughout the analysis of coinfected women. In summary, we have identified a panel of biomarkers that are associated with unresolved HCV infection with and without HIV co-infection. In the HCV RNA+ group, APRI scores, sICAM, sVCAM, and IP-10 all were elevated, pointing to a potential pathway whereby HCV replication drives inflammation and induces hepatic fibrosis. APRI in HCV RNA+ positively correlated with markers of inflammation and higher concentrations of these markers were especially seen in ART-treated subjects with uncontrolled HIV replication implying that co-infection can amplify the pro-inflammatory effects of HCV replication. Finally, a subset of markers was found associated with lower fibrosis and with HIV negative or viremic control and these markers may be important in identifying a better prognosis in Elite or treated individuals. Our findings will inform future investigations into the underlying mechanisms of liver pathogenesis and will reveal inflammatory mediators most relevant to these processes. The clinical implication of these findings is that new, potent anti-HCV therapy may be more effective in reducing the pro-inflammatory environment in those HIV co-infected subjects who have controlled HIV replication, and likely would not significantly reverse the inflammatory state in those with uncontrolled HIV replication. However, anti-HCV therapy would be predicted to halt or slow liver fibrosis, even among subjects with uncontrolled HIV replication.

## Supporting information

S1 Fig Cytokines and chemokines correlate with liver fibrosis. Cytokines, chemokines and cell adhesion molecules were evaluated for correlation with FIB-4, a marker of liver fibrosis. The subgroups were broken down by color: HIV Neg (blue), Elite (green), HIV uncontrolled (ARTuc; red) and HIV controlled (ARTc; orange), and by HCV RNA status: positive (closed circles) and negative (open circles).
(TIF)
