# Supplementary figures and images for: The effect of HIV infection and HCV viremia on inflammatory mediators and hepatic injury—The Women’s Interagency HIV Study
Source: PLoS One. 2017 Sep 13;12(9):e0181004. doi: 10.1371/journal.pone.0181004 (PMC5597129; doi:10.1371/journal.pone.0181004)

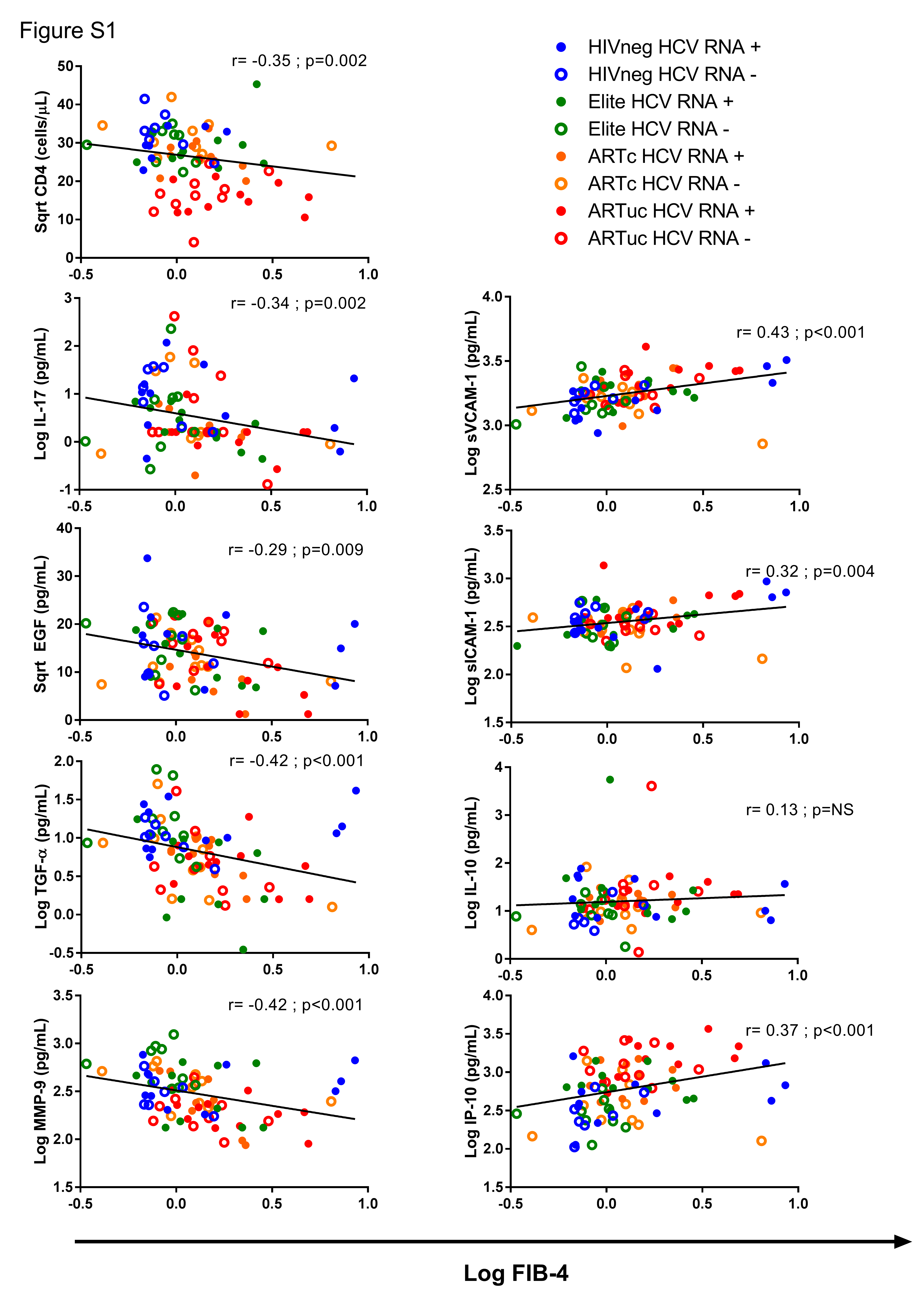

Supplement: S1 Fig — Cytokines, chemokines and cell adhesion molecules were evaluated for correlation with FIB-4, a marker of liver fibrosis. The subgroups were broken down by color: HIV Neg (blue), Elite (green), HIV uncontrolled (ARTuc; red) and HIV controlled (ARTc; orange), and by HCV RNA status: positive (closed circles) and negative (open circles). (TIF) [file pone.0181004.s001.tif]
